# The Biology of Ocular Adnexal Marginal Zone Lymphomas

**DOI:** 10.3390/cancers14051264

**Published:** 2022-02-28

**Authors:** Patricia Johansson, Anja Eckstein, Ralf Küppers

**Affiliations:** 1 Institute of Cell Biology (Cancer Research), Faculty of Medicine, University of Duisburg-Essen, 45147 Essen, Germany; ralf.kueppers@uk-essen.de; 2 Molecular Ophthalmology Group, Department of Ophthalmology, University of Duisburg-Essen, 45147 Essen, Germany; anja.eckstein@uk-essen.de

**Keywords:** ocular adnexal lymphoma, extranodal marginal zone lymphoma, mucosa-associated tissue, MALT lymphoma, NF-κB, ocular adnexa, orbit

## Abstract

**Simple Summary:**

Ocular adnexal marginal zone lymphoma (OAMZL) is a distinct type of lymphoma that presents in tissues around the eyeball. The lymphoma develops from mature B lymphocytes that have been triggered by antigens for prolonged times. It seems that the B cells often recognize autoantigens. The lymphoma cells often carry specific chromosomal gains and, in some cases, chromosomal translocations. A main factor in the development of this lymphoma is the constitutive activation of the NF-κB pathway, which occurs through various types of genetic alterations. Further key pathogenetic mechanisms involve epigenetic changes, indicated by recurrent mutations in epigenetic regulators.

**Abstract:**

This review focuses on the biology of ocular adnexal marginal zone B-cell lymphomas of the mucosa-associated lymphatic tissue (MALT) (OAMZL) subtype. The ocular adnexa includes all structures and tissues within the orbit except for the eye bulb. In the region of the ocular adnexa, MALT lymphomas represent the most common subtype of lymphoma, accounting for around 8% of all non-Hodgkin lymphomas. These lymphomas are often preceded by inflammatory precursor lesions. Either autoantigens or infectious antigens may lead to disease development by functioning as continuous antigenic triggers. This triggering leads to a constitutive activation of the NF-κB signaling pathway. The role of antigenic stimulation in the pathogenesis of OAMZL is supported by the detection of somatic mutations (partially with further intraclonal diversity) in their rearranged immunoglobulin V genes; hence, their derivation from germinal-center-experienced B cells, by a restricted *IGHV* gene usage, and the validation of autoreactivity of the antibodies in selected cases. In the established lymphomas, NF-κB activity is further enforced by mutations in various genes regulating NF-κB activity (e.g., *TNFAIP*3, *MYD88*), as well as recurrent chromosomal translocations affecting NF-κB pathway components in a subset of cases. Further pathogenetic mechanisms include mutations in genes of the NOTCH pathway, and of epigenetic regulators. While gene expression and sequencing studies are available, the role of differential methylation of lymphoma cells, the role of micro-RNAs, and the contribution of the microenvironment remain largely unexplored.

## 1. Definitions

### 1.1. Ocular Adnexa

Adjacent structures of the eye are called the ocular adnexa, which include the content of the orbit, the lacrimal apparatus, the extraocular muscles, the eyelids, the eyelashes, the eyebrows, and the conjunctiva. Most definitions of the orbit include extraocular muscles, blood vessels, nerves, fascia, and fat. The eyeball lying in the cavity is not part of the ocular adnexa. Details on anatomical structures can be found in relevant textbooks [1,2].

### 1.2. Extranodal Marginal Zone Lymphomas

Whereas most B-cell lymphomas primarily locate to the lymph nodes or other lymphoid organs, some lymphomas have a primary presentation in extranodal sites. The predominant form of such lymphomas is extranodal marginal-zone lymphoma (EMZL) [3]. The histological picture resembles marginal zones, the prototype of which is seen in the spleen, and marginal-zone B cells are indeed considered to be the normal counterpart of EMZL. Most EMZLs arise in mucosal tissues; hence, these lymphomas are then also defined as mucosa-associated lymphoid tissue (MALT) lymphomas. EMZL can occur at various locations in the body, including the stomach, the salivary glands, and the ocular adnexa. Ocular adnexal lymphomas (OALs) do not encompass lymphomas developing in the eyeball.

The majority of OALs are EMZLs of the MALT subtype developing in sites lacking germinal-center-containing lymphatic tissues. Primary OALs have to be distinguished from secondary lymphomas caused by involvement of the ocular adnexa with systemic lymphomas. Such systemic lymphomas involving the ocular adnexa are mostly diffuse large B-cell lymphomas, and will not be further considered here. In the following text, we will focus on the biology of ocular adnexal marginal zone lymphomas (OAMZLs). We refer to other recent reviews for clinical, diagnostic, and pathological aspects of OAMZL [4,5,6,7].

## 2. Epidemiology

In the orbit, OALs are the most common malignancy in adulthood, and account for 11% of all orbital masses. Primary OALs are nevertheless rare; they represent only 1–8% of all non-Hodgkin lymphomas [8,9,10], and 5–15% of all extranodal non-Hodgkin lymphomas [8]. The most common subtype of primary lymphoma of the ocular adnexa is extranodal marginal zone lymphoma of the MALT subtype, accounting for approximately 67–80% of cases [11,12]. Geographic differences have been recognized, with around 60% of OALs in the Western world being represented by OAMZLs, whereas in Asian case series approximately 80% of primary OALs are of this type [5,9,11,13]. The next most common lymphoma types in the ocular adnexa region are follicular lymphomas, mantle-cell lymphomas, and diffuse large B-cell lymphomas, each accounting for approximately 10% of primary OALs [11]. Other subtypes of B-cell lymphoma and T-cell lymphoma are very rare in this location. Overall, primary EMZL accounts for up to 74% of all orbital malignancies.

OALs show different frequencies for their sites of involvement. Primary conjunctival and eyelid lymphomas are reported in 20–33% and 5–24% of OALs, respectively [6,14,15]; the lacrimal drainage apparatus is rarely involved. For the lacrimal gland, the annual incidence is about 0.7/million individuals; 99% of lymphomas in this location are B-cell lymphomas, with 68% of them EMZLs. The lacrimal sac is reported to be involved in 2% of patients [16,17]. Secondary involvement of the ocular adnexa with a systemic lymphoma is described in only 10–32% of all OAL cases [18].

From 1975 to 2001, the incidence of OAL has increased by 6.3% annually, which is more rapidly than seen for other extranodal non-Hodgkin lymphomas [19,20]. A nationwide Danish study also reported a rising incidence of OAL from 1980 to 2017. This was observed for OAMZL for men and women, but not for follicular lymphoma, mantle-cell lymphoma, or diffuse large B-cell lymphoma [11]. The reasons for the rising incidence are not known. An increase in the annual incidence of OAMZL from 2001–2017 was also reported by Cerhan et al. for the USA [21].

## 3. Morphology and Immunophenotype

**Figure 1:**
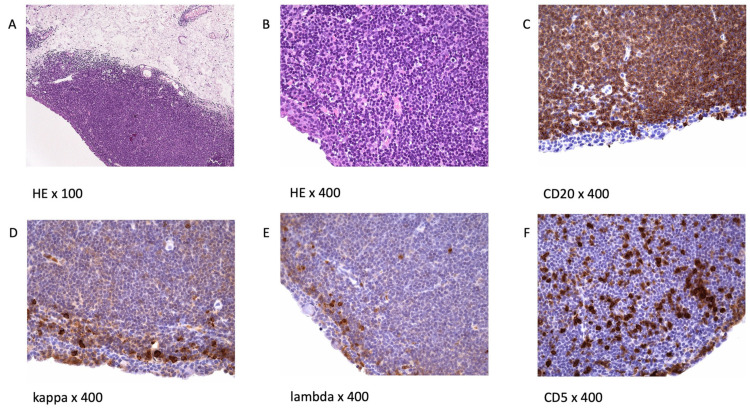
Histology and immunophenotype of OAMZL: (**A**) immunohistochemistry (Hematoxylin and Eosin, H&E staining) of conjunctival lymphoma; (**B**) enlarged view of an H&E staining, demonstrating a dense infiltrate of small, round-shaped cells; (**C**) the lymphoma cells express CD20; (**D**,**E**) in this case, no clear overexpression of one light chain is observed; (**F**) CD5 staining. Tumor cells are negative; the intermingled CD5^+^ cells represent T cells.

Morphologically, OAMZL is characterized by an infiltrate with expansion of the marginal zone surrounding residual germinal centers, if recognizable. The infiltrate consists of a heterogeneous population of small, round-shaped lymphocytes, including centrocyte-like cells, monocytoid cells, and/or scattered immunoblasts (Figure 1) [22]. Plasmacytic differentiation is frequently seen. The plasmacytic cells express plasma cell markers only in 11% of cases [23]. The occurrence of these cells is not significantly associated with recurrence, systemic disease, or lymphoma-related death [23]

Regarding the immunophenotype, OAMZL exhibits the same phenotype as other MALT lymphomas. For identification of a MALT lymphoma, no typical aberrant immunophenotypic markers are available. The cells express CD20, CD79a, PAX5, IgM, BCL2, and TCL1; several cases also express CD11c, CD43, and CD35; rare cases express CD5. The OAMZL cells are negative for IgD, CD3, CD10, CD23, cyclin D1, BCL6, and IRF4. The negativity for CD10, CD23, cyclin D1, and BCL6 can be helpful in the differential diagnosis of OAMZL versus small lymphocytic lymphoma (CD23^+^), mantle-cell lymphoma (cyclin D1^+^), and follicular lymphoma (CD10^+^BCL6^+^) [3]. The immunophenotype of OAMZL lymphoma cells is therefore very similar to that of normal marginal-zone B cells. Light-chain restriction as an indication of monoclonality is not regularly detectable [24]. An example is given in Figure 1.

## 4. Bilateral and Recurrent Disease

Lymphomas diagnosed as primary OAMZL can arise bilaterally, either simultaneously or sequentially. Relapses can occur in nearly the same position, usually not in localizations previously irradiated, or can be found on the other eye. In rare cases, systemic relapses occur. The time between first manifestation and relapse can take more than a decade. Based on *IGHV* clonality analyses, Matsuo et al. observed that bilateral, recurrent, or systemically multifocal lesions represent a common clone [25].

Interestingly, the type of ocular adnexal and systemic lymphoma occurring in one patient can be different. In an analysis of 209 OAL patients—31% of them presenting with a history of systemic lymphoma—the histological subtype was the same in 83% [26]. In 12.3% of patients, the OAL was more indolent than the systemic lymphoma. Several explanations are possible, e.g., transformation, composite lymphomas, or trafficking of cells to tissues encountering different microenvironmental settings. The mechanisms behind this are not yet well investigated. The observation of different lymphoma subtypes underscores the importance of biopsies for determination of the histological subtype, even in the context of systemic lymphoma [26].

## 5. Etiology and Pathogenesis

### 5.1. Precursor Lesions

Several precursor lesions potentially developing to OAMZL have been described, among them orbital pseudotumors (idiopathic orbital inflammatory disease (IOID)), reactive lymphoid hyperplasia (RLH), and IgG4-related disease [27]. Both exogenous antigens and autoantigens can trigger the abovementioned precursor lesions in the ocular adnexa. Precursor lesions as inflammatory non-malignant states have in common that they result in chronic antigenic stimulation, which may lead to activation of the NF-κB signaling pathway, to chromosomal alterations, and to other genetic and epigenetic alterations. This multistep process can drive lymphoma development [28].

OAMZL arising in the context of IgG4-related disease has been repeatedly reported as OAMZL with IgG4-positive cells or infiltrated by IgG4-positive cells [29,30,31,32,33]. In some cases, the differentiation between the two entities is challenging, since OAMZL exhibits IgG4-positive plasma cells in up to 62% of cases [34]. Histopathologically, obliteration of venous vessels is specific. Plasma cells in IgG4-related disease are polytypic. In IgG4-related disease, eosinophilia, high IgE titers, polyclonal hypergammaglobulinemia, and often elevated serum IgG4 levels are observed [35]. Infraorbital nerve enlargement is a unique feature on MRI scans [31]. Upregulation of activation-induced cytidine deaminase (AID)—the master factor for somatic hypermutation and class-switch recombination of immunoglobulin genes—was observed in IgG4-related ophthalmic disease and OAMZL, whereas AID expression was lower in IgG4-negative OAMZL [36]. AID might be a driver for oncogenesis in the development of IgG4-related ophthalmic disease to IgG4-positive OAMZL.

### 5.2. Antigen Stimulation

Chronic (auto)antigenic stimulation via chronic inflammation, infection, or autoimmune disease is supposed to be a relevant pathogenic mechanism in the development of primary MALT lymphoma in general [37]. A multistep process is supposed to promote survival and growth advantages of stimulated B cells, which may finally give rise to monoclonal B-cell populations. Various mechanisms are described causing this antigenic stimulation.

#### 5.2.1. Infectious Agents

##### *Chlamydophila* *psittaci*

*Chlamydia* are human pathogenic intracellular bacteria that are typically transmitted via infected birds. Mostly, infections are asymptomatic, but they can cause pneumonia, chronic conjunctivitis, pericarditis, and hepatitis [38]. *Chlamydophila psittaci* can induce immune reactions cross-reacting with autoantigens, leading to insufficient elimination of the pathogen and induction of lymphoma development [7,39,40].

The prevalence of *C. psittaci* in OAL seems to be region-specific. In most studies involving patients from Italy or Korea, *C. psittaci* was repeatedly detected by PCR and other methods in OAL cases. Other *Chlamydia* species were predominantly observed in China (*C. pneumonia*) and the UK (*C. trachomatis*) [7,41,42]. In other countries, however—including Japan, the USA, Cuba, the UK, the Netherlands, France, and Germany—no evidence for *C. psittaci* in samples of OAL was observed [43,44,45,46,47,48,49,50,51].

(1) Other Bacteria

Among 308 OAL patients analyzed in 11 studies, *Helicobacter pylori* was detected in 23% of the lymphomas [52]. However, there is the risk of contamination of OAL biopsy specimens by the rather prevalent *H. pylori* during sampling, so the true incidence may be substantially lower. Notably, the prevalence of OAL patients with *H. pylori*-positive gastric infections is overall no higher than in the general population [52], arguing against a significantly increased risk of OAL development in individuals with chronic gastric *H. pylori* infection. A recent study confirmed a lack of association between gastric *H. pylori* infection and OAL incidence [53]; none of 18 OAL cases in that study showed *H. pylori* DNA in the lymphoma tissue. Thus, the role of *H. pylori* in the development of OAL is still unresolved.

(2) Viral Pathogens

In several studies on a potential viral etiology of OAL, no viruses were detected in the lymphomas [51,54]. For chronic hepatitis C virus (HCV) infections, the association with marginal-zone lymphomas is especially well known [55,56]. Although there are clear indications for a role of HCV in the pathogenesis of some types of B-cell lymphoma, its role in OAMZL is less clear [57].

For human-immunodeficiency-virus-infected patients, a higher risk of developing marginal-zone lymphomas has been described, but there are no reports on higher incidences of OAMZL [58]. Regarding Epstein–Barr virus, cytomegalovirus, and human papilloma virus, there are also no reports on higher infection rates in patients with OAL [54,59].

#### 5.2.2. Autoimmune Diseases

**Figure 2:**
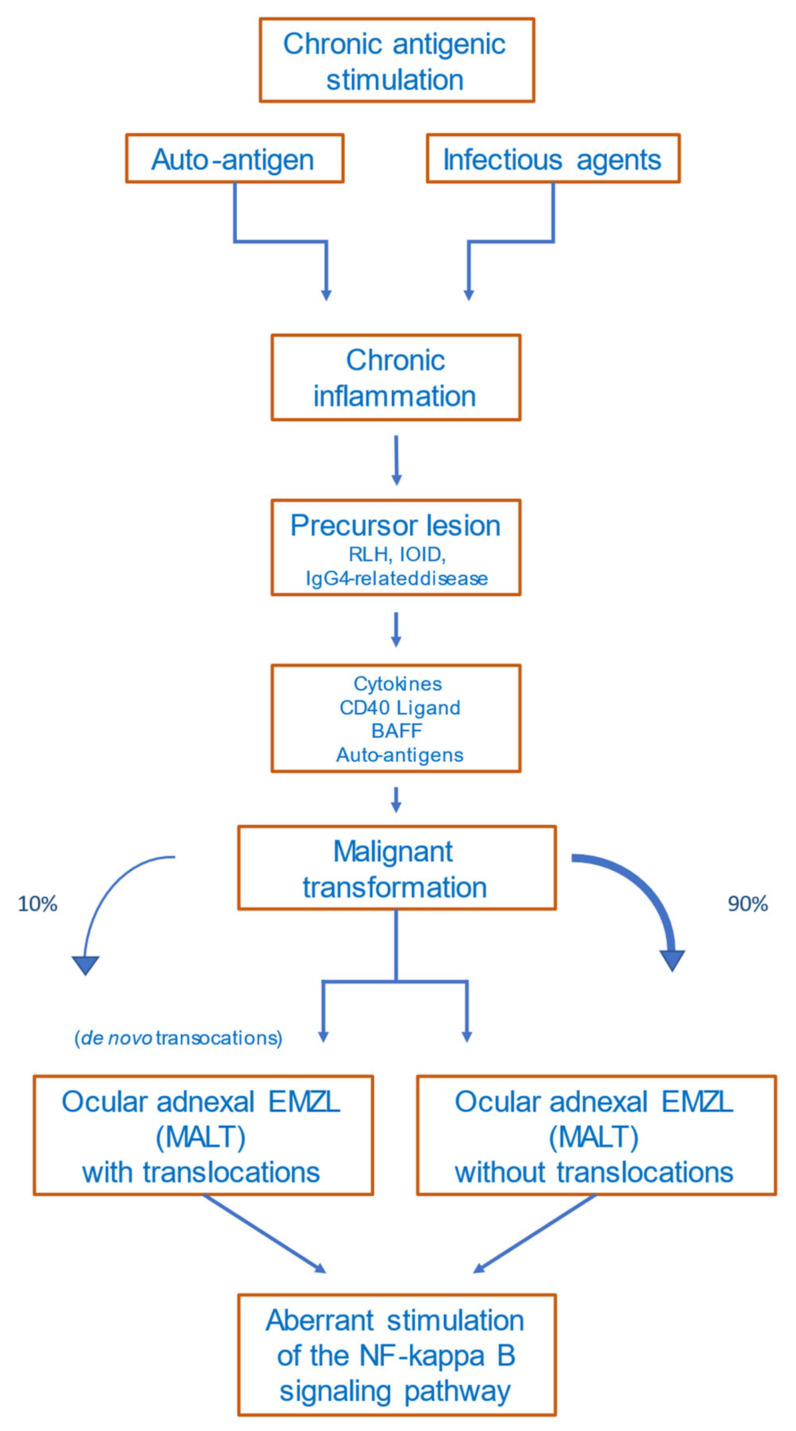
Proposed scheme of OAMZL pathogenesis. RLH: reactive lymphoid hyperplasia; IOID: idiopathic orbital inflammatory disease; BAFF: B-cell activating factor; EMZL: extranodal marginal-zone lymphoma; MALT: mucosa-associated lymphatic tissue.

The detailed mechanisms of lymphomagenesis in the context of autoimmunity remain unclear. The occurrence of lymphomas in association with rheumatoid arthritis, Sjögren’s syndrome, Hashimoto thyroiditis, and other autoimmune diseases is well described [60,61]. A meta-analysis of 20 studies including patients with the abovementioned autoimmune diseases revealed that lymphomas are more common in these patients than in healthy subjects [62]. The most common lymphoma subtype occurring in patients with autoimmune diseases is marginal-zone lymphoma [63]. Disease activity, the presence of rheumatoid factor, and/or cryoglobulinemia in patients with autoimmune diseases are prognostic factors for lymphoma development, reflecting a continuing immune stimulation [64]. Apoptotic resistance—mediated by high BCL2 expression, activation of NF-κB, and overexpression of B-cell activating factor (BAFF)—is increased in autoimmune diseases [65]. Figure 2 provides a proposed scenario of OAMZL pathogenesis.

### 5.3. Chromosomal Aberrations

#### 5.3.1. Translocations

In OAMZL, chromosomal translocations leading to constitutive activation of the NF-κB signaling pathway have been described [22]; these include t(11;18)(q21;q21)/*BIRC3-MALT1*, often accompanied by a trisomy 3 [66], and t(14;18)(q32;q21)/*IGH-MALT1* (Table 1). The t(11;18)(q21;q21) juxtaposes *BIRC3* (previously also known as *API2*) to *MALT1*, resulting in a fusion gene; this translocation has been detected in 10–15% of OAL cases [67,68]. The t(14;18)(q32;q21) translocation brings the *MALT1* gene under control of the IGH locus enhancers, causing constitutive expression of MALT1, and is present in around 5–10% of OAMZLs. MALT1 is a protease and an important mediator of canonical NF-κB signaling [69].

The t(3;14)(p14.1;q32)/*FOXP1-IGH* was observed in around 5–15% of OALs, and leads to constitutive expression of FOXP1 [75,76,88]. FOXP1 is a transcription factor that supports B-cell survival, and can cooperate with NF-κB, so that for this translocation event there is also a link to the NF-κB pathway [89].

Further translocations occur in OAMZL with lower prevalence. These include t(1;14)(p22;q34) juxtaposing *BCL10* to the *IGH* locus, or t(5;11) with unknown translocation partners [90]. Notably, translocations are observed in OAMZL at lower frequencies than in MALT lymphomas occurring in other regions, and the various EMZLs show distinct patterns of recurrent chromosomal translocations [88].

#### 5.3.2. Copy Number Variations

The most frequent copy number variations in OAMZL are trisomy 3 and trisomy 18, in approximately 30–60% and 20–55% of patients, respectively (Table 1) [70,71]. Trisomy 3 is more common in persons above 50 years of age, whereas trisomy 18 is observed mostly in younger, female patients. The cases with trisomy 18 have more lymphoproliferative lesions, less nodularity, and are associated with recurrent disease [91]. The pathogenetic effect of these trisomies in OAMZL is unknown.

### 5.4. Genetic Alterations in Particular Signaling Pathways

#### 5.4.1. Nuclear Factor Kappa B (NF-κB) Pathway

NF-κB is a transcription factor family that plays a critical role in B-cells’ activation, development, and survival [92]. The NF-κB pathway is normally only transiently activated in B cells by binding of various ligands to receptors, including Toll-like receptors, the TNF-α receptor, the BCR, CD40, and others. The intracellular signaling transduction is mediated via a canonical and a non-canonical pathway [88]. A major role of deregulated activation of this pathway in OAMZL is already indicated by the fact that the three most frequent chromosomal translocations of OAMZL cause, or at least contribute to, NF-κB activation, as discussed above. A further major driver for constitutive NF-κB activation in OAMZL is genetic alterations leading to functional changes of TNF-α-inducible protein 3 (TNFAIP3), previously also called A20. Somatic deletions and/or point mutations lead to inactivation of this negative regulator of the classical NF-κB pathway [79]. In OAMZL, destructive *TNFAIP3* mutations and/or deletions were observed in 30–50% of cases [77,80] (Table 1). *TNFAIP3* is the most frequently mutated gene detected so far in OAMZL. It has been reported that *IGH/MALT1* translocations are mutually exclusive with *TNFAIP3* mutations/deletions in OAMZL [79], indicating that these are alternative mechanisms for deregulated NF-κB activation in OAMZL.

To identify potential mutations in further genes of the NF-κB signaling pathway, sequencing analyses were performed by several groups. In 24 OAMZL samples, sequencing of hotspots in the genes *CARD11*, *MYD88*, and *CD79B*, known to be frequently mutated in other B-cell lymphomas, revealed no mutations [93]. In a targeted sequencing approach of genes involved in the NF-κB signaling pathway performed by our team with 63 patients, mutations in *TNFAIP3* (27% of cases), *MYD88* (19%), and *BCL10* (6%) were observed [78]. Further genes, mutated to lower frequencies, were *TNIP1*, *NFKBIA*, *BIRC3*, *CARD11*, and *CD79B*. Only a few genes encoding components of the non-canonical NF-κB pathway were mutated (*MAP3K14*, *BIRC3*, and *CYLD*), whereas other mutated genes were involved in the canonical pathway [78]. A further study analyzing the frequency of *MYD88* L265P mutations in primary OAL found the gene to be mutated in 36% of patients [82]. In a targeted next-generation sequencing (NGS) approach to OAL including 20 samples, with 17 of them being primary OAL, 25% of cases exhibited mutations in the TIR domain of *MYD88* [83]. Two of the three studies mentioned carefully excluded lymphoplasmacytic lymphoma/Waldenström macroglobulinemia by analyzing paraproteins and plasmacytic differentiation—especially with IgM [78,83], which is necessary when analyzing *MYD88* mutations. Further NGS-based approaches using whole genomes, whole exomes, and targeted sequencing confirmed and extended these findings [51,80,86].

#### 5.4.2. NOTCH Pathway

The NOTCH signaling pathway is important in cell differentiation; it is active in many cell types regulating cell development, differentiation, and homeostasis, and is involved in many malignant diseases, including lymphomas [94,95,96]. The NOTCH signaling pathway cross-interacts with the NF-κB signaling pathway as its upstream regulator [97,98,99]. Non-synonymous *NOTCH1* or *NOTCH2* mutations were observed in up to 10% of OAMZLs, with a similar pattern as described for other B-cell lymphomas. There is a clustering of the mutations in the HD and PEST domains of NOTCH1, as well as downstream of the ankyrin repeats in the intracellular domain of NOTCH2 [78,80]. For both genes, these types of mutations cause a gain of function, as the inhibitory C-terminal PEST domains are removed or otherwise inactivated. Copy number gains in the NOTCH target *HES4* may be a further mechanism of enforced NOTCH pathway activity in OAMZL [51,100].

#### 5.4.3. NFAT Signaling

A recent exome sequencing study provided the first evidence for recurrent alterations in the NFAT signaling pathway in OAMZL. Frequent deletions and destructive mutations were detected in the gene encoding the negative NFAT signaling regulator CABIN1 (30% of cases with mutations), as well as rarer mutations in NFAT members themselves and other NFAT signaling pathway components.

### 5.5. Epigenetic Regulators

Alterations in epigenetic modifiers occur in several types of lymphoma [101]; additionally, in OAMZL, genes encoding epigenetic regulators are mutated. Among these genes are *KMT2D* (approximately 5–20% of cases) and *CREBBP* (ca. 15%). In individual studies, mutations in the epigenetic regulators *KMT2C* and *EP300* have been detected, such that a clear determination of the overall frequency of such alterations needs further investigation [78,80,86]. KMT2C and KMT2D, belonging to the mixed-lineage leukemia (MLL) family of histone methyltransferases, methylate Lys-4 of histone H3. Mutations leading to inactivation of these genes lead to diminished global H3K4 methylation in follicular and diffuse large B-cell lymphoma [102]. Conditional deletion of *Kmt2d* in different developmental stages of B cells in mice resulted in an increased number of germinal-center B cells end enhanced proliferation. From these results, the authors suggested the *KMT2D* acts as tumor suppressor gene. KMT2C, which functions very similarly to KMT2D, and can partially replace a loss of KMT2D, is therefore also supposed to be tumorigenic in case of a loss [103]. CREBBP and EP300 are related histone and non-histone acetyltransferases, which regulate transcriptional activity in several signaling pathways via chromatin remodeling. In follicular and diffuse large B-cell lymphoma, monoallelic deletions/mutations result in defects of acetylation of the oncoprotein BCL6 and the tumor suppressor p53.

### 5.6. Additional Mutated Genes

In addition to the genes in major signaling pathways or involving epigenetic regulators already mentioned, further genes recurrently mutated in OAMZL include *TBL1XR1* [81]. *TBL1XR1* is an essential regulator of transcriptional repression, and contributes to canonical NF-κB activation [104]. This gene can activate the transcription of transcription factors such as NF-κB and JUN [105], and may therefore contribute to the strong NF-κB activity in OAML. *TBL1XR1* is mutated in various tumors and lymphomas, promoting tumor cell survival. In OAMZL, mutations were consistently detected in 10–20% of cases, so *TBL1XR1* is one of the most frequently mutated genes in this type of lymphoma (Table 1). *TBL1XR1* mutations have been linked to a poor prognosis in aggressive lymphomas [106,107]. In a small cohort of patients with OAL of the MALT subtype, *TBL1XR1* mutations were associated with unique morphometric phenotypes [108]; the cells exhibited significantly lower circularity and solidity as analyzed via computational digital image analysis.

The JAK/STAT signaling pathway is necessary for cytokine signaling and immune regulation, and plays an important role in various types of lymphoma [109]. Activating mutations in *JAK3*, known to cause constitutive activation of the JAK/STAT signaling pathway, were observed in up to 10% of patients with OAMZL [51]. Interestingly, patients with mutant *JAK3* exhibited a shorter progression-free survival [51].

In a recent study, deletions and non-synonymous mutations in the *RHOA* gene were detected in 26% of OAMZL studies [87]; this points to a potential role of altered Rho signaling in OAMZL, but further studies are needed in order to clarify the consequences of the mutations detected in *RHOA*.

## 6. B-Cell Receptors of OAL

Analysis of the rearranged immunoglobulin genes of B-cell lymphomas can reveal valuable insights into the specific B-cell subset from which a lymphoma derives. Moreover, in various types of B-cell lymphoma, the BCRs of the lymphoma cells recognize foreign or autoantigens, which may represent a major factor in lymphoma’s pathogenesis [110]. Numerous studies of the IGHV genes of OAL—mostly those of the MALT type—showed that in nearly all cases the lymphoma cells carried somatically mutated IGHV genes [76,111,112,113,114,115,116]; this revealed the origin of OAL from germinal-center-experienced B cells, as the process of somatic hypermutation, which produces such mutations, takes place specifically in germinal-center B cells [117]. In a substantial fraction of cases, there were signs of low-level ongoing hypermutation, indicating that the lymphoma cells had retained some features of germinal-center B cells, and may still be antigen-driven [76,112,113,116]. A biased IGHV gene usage can be a further indication that the BCRs of a particular type of lymphoma are specific to a limited set of antigens. Numerous studies of OAMZL have addressed this issue and, indeed, revealed that some IGHV genes were more frequently used than expected based on their usage in the normal B-cell population. The fact that not all studies found the same biased IGHV gene usage may be due to the often rather small number of cases studied, and the fact that most studies involved cases from distinct continents, suggesting that geographic conditions may influence IGHV gene usage. The IGHV genes repeatedly reported to be frequently rearranged by OAL clones include *IGHV4-34*, *IGHV3-7*, *IGHV3-23*, and *IGHV3-30* [76,112,113,115,116,118]. Several of these genes are known to be frequently used by autoantibodies, raising the suspicion that the BCRs of OAL may often be autoreactive; this is also implied by the observed link between these lymphomas and autoimmune diseases, as discussed above. This hypothesis was experimentally validated by showing autoreactivity of five out of five antibodies tested [119].

Taken together, the numerous studies on the BCR sequence features have provided strong indication that OAL is derived from antigen-experienced (post) germinal-center B cells that have a biased IGHV usage and are often autoreactive. The chronic stimulation of such autoreactive B cells may be an important factor in the transformation of these cells into malignant OAL clones.

**Figure 3:**
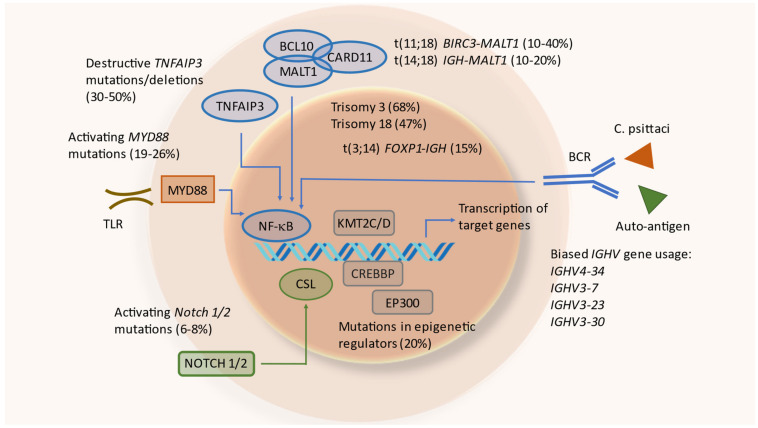
Mechanisms and pathways involved in the pathogenesis of OAMZL. TLR: toll-like receptor; *C. psittaci*: Chlamydophila psittaci.

The mechanisms and pathways involved in the pathogenesis of OAMZL are depicted in Figure 3.

## 7. Altered DNA Methylation

Altered DNA methylation plays a significant role in the pathogenesis of marginal-zone lymphomas [120]. Promoter methylation in *TNFAIP3*—which is also frequently mutated in OAMZL, as discussed above—was observed in 19% of OAMZLs, and was associated with hemizygous deletion of *TNFAIP3*, but mutually exclusive with mutations in the gene. Promoter methylation seems to have an additive effect in the regulation of A20 expression, since promoter methylation in addition to hemizygous deletion resulted in lower transcript expression [79].

Choung et al. investigated aberrant promotor methylation of known or suspected tumor-suppressor genes in OAMZL, as well as the potential association with *C. psittaci* infections in 35 cases [121]; they observed CpG island methylation in nine genes, which was not the case in any of the 13 control cases. *C. psittaci* was detected in 75% of cases available for analysis. *ECAD* hypermethylation was significantly higher in *C. psittaci*-positive cases, but was not correlated with clinical characteristics. A comparison of genome-wide DNA methylation profiles in relation to *C. psittaci* infection status and response to doxycycline treatment in OAMZL revealed distinct methylation patterns. The methylation status of *DUSP22* was likely attributable to infection, whereas methylation of the genes *IRAK1* and *CXCL6* may reflect treatment response [122].

## 8. Altered microRNA Expression

The role of microRNAs (miRNAs) in OAMZL is not well understood. Hother et al. studied miRNA expression via microarray analysis in a cohort of 18 OAMZLs, 15 of them being primary lymphomas. Comparing the results to aggressive lymphomas, the authors observed MYC- and NFKB1-mediated dysregulation of miRNA expression in the samples with aggressive lymphomas; they concluded the existence of fundamental differences in miRNA expression between OAMZLs and diffuse large B-cell lymphomas, mainly due to differences in the MYC and NF-κB regulatory pathways [123].

## 9. Microenvironment

In the absence of a local trigger such as *C. psittaci*, it is unclear why EMZLs arise in the specific location of the ocular adnexa. The ocular adnexa lack lymphatic tissue, which contains germinal centers. Lymphocytes therefore have to migrate and home to the ocular adnexa. In an immunohistochemical analysis of 91 lymphoma cases, including 28 OAMZLs, homing and trafficking of lymphocytes by chemokines, sphingosine-1-phosphate, chemokine receptors, and integrins was analyzed [124]. Comparing lymphomas arising in secondary lymphatic organs with OALs, distinct patterns of lymphocyte homing molecules were observed. Analysis of molecules responsible for entry in extranodal sites—such as CXCR4, CXCL12, and α4β1 integrin—revealed a high CXCR4 expression for EMZL. CXCL12 expression was higher for extranodal lymphomas than for nodal lymphomas, providing a strong homing signal. Retention in the tissue was mediated by CXCR5 and CXCL13 expression [124].

The role of a specific microenvironment in OAMZL needs to be further analyzed. Kim et al. analyzed tumor-infiltrating T cells [125]; their group was particularly interested in the clinical significance of tumor-infiltrating FOXP3^+^ T cells in patients with OAMZL. Biopsy specimens of 42 patients with and without treatment were stained for FOXP3 expression. Treatment was chemotherapy, radiotherapy, doxycycline, or a watch and wait concept. A complete response was observed in 50% of patients. A high number of FOXP3^+^ T cells was associated with a higher rate of complete response and a tendency towards a prolonged progression-free survival [125].

In 2013, Kinoshita et al. investigated the role of the vascular endothelial growth factor (VEGF) in OAMZL and RLH. In 22 OAMZLs and 16 reactive samples the VEGF and CD20 positivity were higher in the lymphomas than in the RLH samples. Microvessel density as determined by anti-CD34 staining showed a significant positive correlation with VEGF/CD20 expression. The authors suggested a significant role for VEGF in the pathogenesis and tumor angiogenesis of OAMZL [126].

## 10. Conclusions

There is now compelling indication that continuous antigenic stimulation of B cells by particular bacterial infections or autoantigens is the basis for the development of OAMZL. Such an initial and prolonged antigenic triggering of B cells is a general feature of marginal-zone B-cell lymphomas, as well as several other types of B-cell lymphoma [110]. Although the lymphoma is located outside the secondary lymphoid organs, it is obvious that a T-cell-dependent germinal-center reaction plays an essential role in the pathogenesis of lymphoma, as nearly all cases carry somatically mutated IGV genes, representing a genetic trait of a germinal-center reaction. This reaction likely plays a role in the pathogenesis of OAMZL not only by generating long-lived and presumably high-affinity B cells, but also by promoting chromosomal translocations and mutations in non-IG genes [127]. OAMZL therefore belongs to the large group of germinal-center-derived B-cell lymphomas [127,128]. A further similarity not only to other marginal-zone B-cell lymphomas, but also to various other lymphoma types (e.g., many diffuse large B-cell lymphomas and Hodgkin lymphoma), is the major role of constitutive NF-κB activity, mediated by various types of chromosomal translocations and mutations in proto-oncogenes and tumor-suppressor genes [129]. As other genetic alterations detected in OAMZL have also been found in other types of lymphomas, there is (so far) no distinct genetic or epigenetic event known that is specific to only this lymphoma. It is likely that it is the particular combination of antigenic specificities of the lymphoma cells, microenvironmental interactions, and genetic and epigenetic alterations that specifies OAMZL as a distinct type of human B-cell lymphoma.

## Figures and Tables

**Table 1:** Genetic alterations of OAMZL.

Chromosomes or Genes Affected	Type of Genetic Alteration	Pathway or Main Function	Approximate Frequency (%)	References
Chromosomal alterations				
Trisomy 3	Chromosomal gain	unclear (FOXP1?)	30–60	[70,71,72]
Trisomy 18	Chromosomal gain	unclear	20–55	[70,71,72]
t(11;18)(q21;q21)	*BIRC3*-*MALT1* translocation	NF-κB pathway	10–15	[68,73]
t(14;18)(q32;q21)	IGH-*MALT1* translocation	NF-κB pathway	5–10	[67,70,74]
t(3;14)(p14.1;q32)	*FOXP1*-IGH translocation	B-cell development and survival (NF-κB pathway)	5–15	[75,76]
Gene mutations				
*TNFAIP3*	Deletions, non-synonymous mutations	NF-κB pathway	30–50	[77,78,79,80,81]
*MYD88*	Non-synonymous mutations (mostly p.L265P)	NF-κB pathway	5–35	[78,81,82,83,84]
*NOTCH1*	Non-synonymous mutations (mostly HD and PEST domains)	NOTCH pathway	2–10	[78,80,85]
*NOTCH2*	Non-synonymous mutations (mostly TAD and PEST domains)	NOTCH pathway	5–10	[78,86]
*KMT2D*	Non-synonymous mutations	Epigenetic regulation	5–20	[78,80,81,86]
*CREBBP*	Non-synonymous mutations	Epigenetic regulation	15	[51,80]
*TBL1XR1*	Non-synonymous mutations (mostly WD40 domain)	Regulation of nuclear receptor activity (NF-κB and AP1 pathway)	10–20	[51,80,81,85]
*JAK3*	Non-synonymous mutations	JAK/STAT signaling	5–10	[51,81]
*CABIN1*	deletions, Non-synonymous mutations	NFAT signaling	30%	[87]
*RHOA*	deletions, Non-synonymous mutations	Rho signaling	26%	[87]

Non-synonymous mutations: includes damaging point mutations, small insertions/deletions.
